# Professionals’ accounts of genetic testing in adoption: a qualitative study

**DOI:** 10.1136/archdischild-2019-316911

**Published:** 2019-07-11

**Authors:** Michael Arribas-Ayllon, Angus Clarke, Katherine Shelton

**Affiliations:** 1 School of Social Sciences, Cardiff University, Cardiff, Wales, UK; 2 Medical Genetics, University of Wales College of Medicine, Cardiff, UK; 3 School of Psychology, Cardiff University, Cardiff, Wales, UK

**Keywords:** children’s rights, comm child health, social work, genetics

## Abstract

**Objective:**

To explore social workers’ and medical advisors’ accounts of genetic testing in adoption.

**Methods:**

A qualitative study using semi-structured interviews to gather in-depth accounts of retrospective cases. Data were analysed thematically to identify professionals’ knowledge and expectations.

**Results:**

Twenty professionals working in adoption services (including 8 medical advisors and 12 social workers) participated in this study. Social workers adopted an essentialist (single-gene) model to discuss genetic testing in relation to past cases. They assumed that testing was a generic procedure for detecting the presence or absence of a specific aetiology, the results of which were believed to be definitive and mutually exclusive. By contrast, medical advisors were circumspect and agnostic about the meaning of results, especially in relation to chromosomal microarray testing. Whereas social workers believed that genetic testing provided clarity in assessment and therefore assisted adoption, medical advisors emphasised the uncertainties of testing and the possibility that prospective adopters might be misled. Medical advisors also reported inappropriate requests to test children where there was a family history of a genetic condition, or to confirm or exclude a diagnosis of fetal alcohol spectrum disorder in children presenting with non-specific dysmorphic features.

**Conclusion:**

Recent advances in genetic technologies are changing the ways in which professionals understand and tolerate uncertainty in adoption. Social workers and medical advisors have different understandings and expectations about the clinical utility of genetic testing. These findings have implications for social work training about genetic testing and enabling effective communication between professional groups.

**What is already known on this topic?**

- Looked-after children are a diverse paediatric population that present with complex physical, developmental and/or health-related problems.
- While recent advances in genome-wide testing have increased diagnostic yield, they also pose new challenges to paediatrics and adoption.
- Clinical significance of chromosomal abnormalities is not well understood, which raises concerns that its detection among looked-after children can be misleading and potentially stigmatising.

**What this study adds?**

- Interviews with social workers and medical advisors reveal different understandings and expectations about the clinical utility of genetic testing in adoption.
- Medical advisors were circumspect and agnostic about the meaning of results, while social workers believed that genetic testing provided clarity and therefore assisted adoption.
- This study suggests that targeted training would assist social work and medical professionals to communicate more effectively about the use of genetic technologies in adoption.

## Introduction

Adopted children are a diverse paediatric population. Many present with complex physical, developmental and/or health-related problems.^1–4^ For each child entering the looked-after system, and for whom adoption is the agreed plan, an initial health assessment by a medical advisor is a statutory requirement.

There are two circumstances in which assessment may involve clinical genetic assessment. First is when a child has a clinical problem or physical features that suggest the condition may be genetic in origin. Paediatricians and clinical geneticists may agree that a child can be tested to obtain a diagnosis to account for their problem. The second circumstance arises when a looked-after child has a family history of a genetic disorder that is not apparent but which that child might either develop later or transmit to their own children in the future. Such circumstances can be further divided into clinical assessment that may involve carrier or predictive genetic testing.

Carrier testing is usually carried out on unaffected individuals at risk of recessive or sex-linked conditions, such as cystic fibrosis or haemophilia. While there may not be any direct health implications, a positive result will affect future reproductive decisions. Predictive testing applies to autosomal dominant conditions usually with late onset, such as Huntington’s disease. Testing positive means the individual will develop the disorder, although onset is variable. In such cases, the general consensus in the genetics community is that children should not be tested unless there is a clear medical benefit in doing so. If there is no medical benefit, then children should not be tested because it removes their autonomy and their right to an open future. The recommendation is that children should be given the choice of genetic testing when they are capable of making their own decisions.^5–8^

The recent introduction of next-generation sequencing technologies has substantially increased both the coverage and resolution of genetic information. While genetic testing may refer to relatively targeted investigations involving the search for mutations in one or more specific genes, ‘genome-wide’ testing can identify multiple variants across the entire genome. Recent advances in diagnostic testing pose challenges to paediatrics and adoption as new methods—microarray-based comparative genomic hybridisation (aCGH)—have largely replaced the light microscopy approach to chromosome analysis as the first-line investigation for identifying a likely cause of a child’s developmental, learning and behavioural difficulties.^9 10^ Microarray can be used to quickly scan a genome for chromosomal imbalances at a very high level of resolution.^11^ The increased sensitivity of detecting small deletions and duplications (collectively known as copy number variations (CNVs)) has improved diagnostic yield from 3% in traditional karyotype testing to approximately 10%–15% in aCGH.^10 12^ It also means that for a significant proportion of cases, diagnostic testing still fails to identify a genetic cause, which challenges the widely held assumption that genetic testing is definitive and straightforward.^9^ In fact, genome-wide screening can produce four types of outcome: finding a diagnosis for the clinical problem, finding no abnormality, finding something of unknown significance or finding an abnormality of likely clinical importance but not causing the clinical problem at hand (known as an incidental finding).^13^

The clinical utility of diagnostic genetic testing is further clouded by the fact that a negative result does not unequivocally rule out a genetic cause because aCGH can only detect copy number changes—it cannot detect changes (variants) within genes.^14^ Moreover, the detection of a variant in a child or parent does not mean it has necessarily caused the specific problem because the clinical significance of some CNVs is not well understood. For instance, a microdeletion within 15q11.2 is commonly associated with a wide range of features including autistic traits and learning difficulties.^15^ However, variable expressivity and incomplete penetrance in populations suggests that this microdeletion is not necessarily causal, which has raised concerns that its detection among looked-after children can be misleading and potentially stigmatising.^16^

Fetal alcohol spectrum disorder (FASD) is a common embryopathy found more frequently among looked-after children.^17^ Clinical diagnostic criteria include evidence of maternal alcohol, intellectual disability, growth restriction and dysmorphic facial features. Diagnosis is challenging, however, because there is no reliable biomarker, clinical features are non-specific and there may be insufficient information about the pregnancy. Futhermore, the associated dysmorphic features overlap with features found in some chromosomal disorders.^9^ For these reasons, the use of aCGH is a valid attempt to exclude other possible causes of developmental problems and dysmorphic features, but the absence of an abnormality is not a direct confirmation of FASD.^18^ The British Medical Association guidelines support the involvement of clinical genetics in gathering evidence of perinatal history and performing careful examination of dysmorphic features before proceeding with genetic testing.^19^

As chromosomal microarray testing becomes a mainstream diagnostic technology, there are concerns that non-genetics professionals, especially those working within social services, may not be fully informed about the limitations of a genetic test result.^16^ Some may believe that genetic testing supersedes clinical assessment and removes uncertainty about a child’s present or future health. The aim of this study was to explore the knowledge and expectations of social workers’ and medical advisors’ accounts of genetic testing in adoption.

## Methods

Twenty participants were recruited through purposive sampling of social workers (n=8), social work managers (n=4) and medical advisors/community paediatricians (n=8) working in adoption services in Wales. Social workers were contacted through the National Adoption Service, all of whom were self-selecting as having first-hand experience of cases that involved genetic testing. Medical advisors working in each of the local authorities were contacted individually via email. Snowball sampling techniques were used to contact specific participants who were singled out as having relevant experience; this widened our recruitment to England. Medical advisors were all mid-to-late career, social workers were more diverse, ranging from the newly qualified to those with over 30 years’ experience. Table 1 presents participant characteristics including the number of cases they discussed in relation to genetic testing. The asterisk (*) symbol indicates where participants discussed cases generically.

**Table 1:** Participant characteristics

Social workers					
Code	Role	Gender	Experience	No. of cases	Location
SWM01	Social work manager	Female	27 years	1	West Wales
SWM02	Social work manager	Female	16 years	3	South Wales
SWM03	Social work manager	Female	22 years	*	South Wales
SWM04	Social work manager	Male	+20	1	London
PAS01	Postadoption support	Female	+20	2	South Wales
CSW01	Child social worker	Female	1 year	1	West Wales
CSW02	Child social worker	Male	14 years	5	South Wales
CSW03	Child social worker	Male	4 years	1	West Wales
CSW04	Child social worker	Female	14 years	3	West Wales
CSW05	Child social worker	Female	+30 years	2	South Wales
CSW06	Child social worker	Male	4 years	1	South Wales
CSW07	Child social worker	Female	2 years	1	West Wales
Medical advisors					
MA01	Community paediatrician	Female	–	3*	Midlands, England
MA02	Paediatric consultant	Male	14 years	5*	South West Wales
MA03	Medical advisor	Female	26 years	6*	West Wales
MA04	Community paediatrician	Female	–	2*	South Wales
MA05	Medical advisor	Female	–	5*	South Wales
MA06	Designated doctor	Female	20 years	9*	South Wales

Semi-structured qualitative interviews were used to gather detailed accounts of retrospective cases. Treating research interviews as ‘accounts’ recognises that interviews are more than representations of past events and experiences, but complex social activities oriented to performing certain actions in the present, such as explaining or defending the speaker’s conduct or practical reasoning.^20^ Interviews explored a range of issues concerning: past cases of preadoption genetic testing, circumstances that trigger a genetic investigation, procedures for recording and communicating genetic information between professionals, practices of sharing information with prospective adopters, the impact of genetic information on postplacement experiences and whether a genetic diagnosis disadvantages a child’s prospects for adoption. All the interviews lasted between 60 and 90 min, were audio-recorded and subsequently transcribed verbatim.

Transcripts were coded via an iterative process of reading and noticing relevant phenomena, allowing the analyst (first author) to arrange data according to differences, commonalities and structures. Coded selections of data were compiled into a subcorpus for group discussion (involving all three authors). Data extracts were then selected in order to identify and illustrate ‘themes’ relating to implicit assumptions and expectations that underpin professionals’ accounts of genetic testing. Each of the major themes represent a range of statements about the reasons given for, and presumed outcomes of, genetic testing in adoption (tables 2 and 3).

**Table 2:** Social workers’ accounts of genetic testing in adoption

Themes	Illustrative accounts
1. *Genetic testing offers definitive and mutually exclusive outcomes*	
*(a) A normal result means that a child does not have a genetic disorder*	SW: So I know she had a genetic test… I: But what happened with that? What was the result? SW: I think it came out fine. I: What do you mean by fine? SW: Well, no genetic abnormality found. So we had to sort of then look for a family who would take a child with developmental delay. (CSW05)
*(b) A result offers clarity about the child’s genetic status*	I think, mostly, the social workers take their lead from the medical advisors. But I suppose you could be in a situation where social workers are finding it hard to find a placement for a child and feel that if they had a genetic test then it would be more clear, and some families might feel that it could be something that they could deal with. (PAS01)
*(c) Genetic testing can reassure prospective adopters*	I think it reassured the adoptive parents (…) I think that now that the testing has taken place and that nothing was found, again that’s offered them some reassurance that, okay, you know, there isn’t a genetic reason for this, the delay. (CSW04)
2. *Genetic testing can confirm or exclude a specific aetiology*	
*(a) Genetic testing can confirm or exclude a diagnosis if there is a family history of a genetic condition*	I think it’s easier when you’re trying to confirm a specific thing actually, because you’re looking for a very specific issue which you can then rule out or in. (CSW03) We tend to use it to eliminate something, like the blood condition. You know, that’s how we’ve seen them used, to eliminate a particular thing. (CSW03) (the medical advisor) felt that (diagnostic testing) would be intrusive and that it wasn’t something that she would be pursuing or recommending. However, we did pursue it. The paediatrician saw the child and I think the foster carers were more observant and felt maybe possibly there was a slight delay with the speech. I think they were looking possibly very much under the microscope and ordinarily wouldn’t have picked up on these issues because they weren’t significant. However, the little girl was eventually tested and indeed she did have the same chromosome issue as her older sister. (CSW05)
*(b) Genetic testing can confirm or* *exclude a diagnosis of FASD*	So it’s not a routine thing but it’s something that social workers always have in the back of their minds. And our children have regular medicals via an adoption medical or looked-after medical, so they’re starting to look now for foetal alcohol (syndrome). I think that’s becoming more possible to test for now. (SWM02)
*(c) Genetic testing investigates a specific aetiology*	I signed consent for a genetic test a little while ago which had been court ordered (^^^) because again of the lifelong implications we are always very very careful about what we sign for, but the court had ordered it so, on that basis I have to sign for it, I have to sign consent as the corporate parent um (pause) but in terms of informed consent what I believed I was signing for was an investigation in relation to a specific condition, now what you’ve told me there that actually that could have thrown anything up, I was consenting to one investigation not an investigation of various different things, so that’s a really interesting point, that something I need to consider, because what am I actually consenting to. (SWM03)
3. *Genetic testing can facilitate adoption*	The more information you’re able to give then the better. And I would say that the genetic testing would fall within that bracket (CSW04) There’s a possibility that being able to test might make it possible to find a family whereas not being able to test the uncertainty might make it very unlikely that you would find a family. (CSW02) I: So as the family finder, if the test had been done it would have helped the— SW: It would have helped me. I feel so, definitely. I know a lot of people don’t agree with that and geneticists usually don’t, but for me I’ve found it’s much better to know than not know. I: Right, even if the tests were positive that the child has a difficulty? SW: Yeah. I: That still makes it easier to place? SW: Yes, definitely. (CSW05) I don’t know if this is a possibility but I suppose if you’re looking at routinely genetically testing every child where there weren’t any issues or concerns it could disadvantage them, couldn’t it? If they are revealing things that you perhaps wouldn’t have worried about before. But I think for children where there are already concerns there, then I think having a diagnosis can only help them really. (CSW06)

FASD, fetal alcohol spectrum disorder; SW, social worker.

**Table 3:** Medical advisors’ accounts of genetic testing in adoption

Themes	Illustrative statement(s)
1. *Genetic testing is neither definitive nor deterministic*	
(a) *A ‘normal’ result does not necessarily exclude a genetic disorder*	And that’s the way that we do explain it is that we all have something or there may be something in all of us that we don’t have any idea what it means at this point in time but in the future there may be more information. And I think it’s trying to explain that, you know, genetics is moving so fast isn’t it that what we have one day is not necessarily, you know, what is normal one day might not be normal the next. (MA04)
(b) *A ‘positive’ result does not necessarily confirm a genetic disorder*	I think you’ve got to be very careful about testing children just because we can. I think you have to think carefully about what a positive result would mean, and I think you mentioned the fact that you can see phenotypic variability. So even if we test a very young child because we can, based upon a family history, and we find a positive result, in terms of then predicting what this is going to mean for the child, I think that’s extremely difficult. (MA02) Yes I have had one (VUSs) where I have got the results the day before panel, the adopters knew it was coming but it was something that was unknown significance and all I did was ask the panel if I could have time to talk to them before they came into panel to explain that to them. And it is like anybody else, you know, it’s sort of saying that there is this, we don’t know what it means, nobody knows what it means at this point of time, it may mean something or nothing. (MA04)
(c) *Adopters and social workers have unrealistic expectations of genetic testing*	And that’s the other thing that we do tell them (social workers) is when we are doing these (tests) is that, you know, we might be looking for something and we pick up something completely different (…) And what all of that means is very difficult and I think they think it’s much more simple. (MA04) So these are the two (cases of genetic testing) where the social worker wanted (it) because (for) some unknown reason, in their head, they’re thinking if all the boxes are ticked that they can get an adopter. And my question is an adopter should accept the unknown. When you have a birth child, you will accept the unknown and so is the unknown because we can’t give answers for all the questions. (MA03) But I think there is this expectation that we can sort of give adopters as much, and rightly so, as much information as we can. But sometimes that is about, yes there is this risk and trying to quantify that risk and to help them to learn to live with that risk rather than saying, yes it is or no it’s not and I think that’s the difficult thing. (MA04) I think a lot of the local authority members and a lot of adoption panel members felt that the children in this family should be tested because if they were tested negative it would all be fine. And what they couldn’t quite understand was, yes but if they are tested and positive the implication for that child and that child has now the choice of knowing has been taken away from them and it’s sometimes difficult for lay people to understand why we do or we don’t do things. (MA04)
2. *Genetic testing is inappropriate*:	
(a) *if the child is unaffected but there is a family history of genetic risk*	I have dealt with a few issues in (place) where the father had said he had Noonan syndrome and the child didn’t have any evidence. And the child did not have and the social worker wanted this child to be tested genetically and I didn’t think it was appropriate so I dug my heel in. I said, tell me what you going to do with that? The child does not have any symptoms and why should I do it? (…) The solicitor spoke to me and I said look there is no signs, there’s no evidence of cardiac problem, any other problem. She might have learning difficulties but the mother and father has learning difficulties and for me, just tell me where the father’s diagnosis was made. (MA03) One (case) I can think of is where there has been a family history of Charcot-Marie-Tooth where there has been huge pressure from the local authority to undertake genetic testing and we are, it’s felt, and in discussion with genetics, it’s not appropriate to do so because the child themselves have got no symptoms. There is nothing we are going to do about it, we aren’t going to treat anything at this point in time, and that the testing should be done at a later date if it is necessary. (MA04)
(b) *to confirm or exclude a diagnosis of FASD*	We’re having requests for children to be tested for conditions. For example, recently, I’ve heard that guardian was pushing for an expert assessment of a child, who she felt needed to be tested for foetal alcohol syndrome, which clearly—well there is no clinical investigation that’s going to give us than answer. Obviously, there is an examination we can do, there’s a history we can consider, but I had seen that child for an adoption medical and it was clear to me that that child didn’t have foetal alcohol syndrome. Whether there might be foetal alcohol effects further down the line, that’s another factor if there were concerns about the mum drinking, but there was certainly nothing that I saw that would warrant any further assessment but the guardian had pushed for, this child needs a test. (MA02) That is what I heard, somebody was saying that one particular judge was demanding genetic testing for foetal alcohol syndrome. Not here, I didn’t have it. (…) one of the doctors did mention that they were asked to do a genetic test for foetal alcohol syndrome and that was demanded by the judge. So we were discussing about educating the judges and then somebody said they had the difficult group to educate. (Laughs) (MA03) I couldn’t quite quantify it exactly but a lot of these cases we will do them on are babies where their mums have been drinking during pregnancy and they have got dysmorphic features and we are looking to exclude other courses other than foetal alcohol, and that’s a big one for us. (…) we are trying to exclude other causes of the dysmorphic features and therefore because, you know, at this point in time we can’t diagnose foetal alcohol syndrome directly it’s let’s exclude everything else, so that is the reasoning behind it. (MA04)
3. *Genetic testing can complicate or hinder adoption*	I think it’s quite a challenge for them (social workers) to understand then the pros and the cons of genetic testing because clearly their thought is around finding an adoptive placement for a child and doing what they can to facilitate that, and that might be a thought that well if we arrange genetic testing, particularly if a test result is negative, well that might be helpful in terms of then a child finding adopters. I guess the flip side of that is well what happens if the test result is positive? Might that make it more difficult, and who is this test for? Is this a test for the adopters or is this a test for the child? (MA02)

FASD, fetal alcohol spectrum disorder.

## Results

### ‘Genetic testing offers definitive and mutually exclusive outcomes’

Social workers often described ‘genetic testing’ as a generic technical procedure, and were generally unaware of recent advances in genome-wide (microarray) testing. A significant finding of the study was that social workers employed a single-gene model to discuss the outcomes of testing in relation to past adoption cases. They assumed that testing was a procedure for detecting the presence or absence of a specific aetiology, the outcomes of which were definitive and mutually exclusive.

#### A normal result means that a child does not have a genetic disorder

When social workers described cases of children referred for diagnostic testing, they interpreted the results of an investigation as a clear and definite outcome. For instance, a normal result was thought to discount genetic factors and imply non-genetic reasons for a child’s difficulties: *“no genetic abnormality found. So we had to sort of then look for a family who would take a child with developmental delay*”.

Several medical advisors reported that children referred for microarray testing often receive normal results. Rather than discounting a genetic explanation, advisors were more agnostic about the meaning of results: *“what is normal one day might not be normal the next”*. A normal result was relative to the limitations or sensitivity of the technology. Only after considering family history and clinical judgement did medical advisors infer non-genetic causes.

#### A (positive) result offers clarity about the child’s genetic status

Some social workers described cases in which a positive result offered clarity about the child’s genetic status. For instance, the detection of a chromosomal abnormality was described as a specific aetiology that *explained* the child’s condition. While in some cases that would be true, in other cases social workers conceded that the clarity of the result was offset by variability of the phenotype, that is, it was uncertain “*how the disorder would manifest itself*”.

Medical advisors were far more circumspect about a positive result. The detection of a chromosomal abnormality was not a *definitive answer*, as one advisor put it. Variants found in the general population do not mean that its detection in an individual was necessarily pathogenic: *“there are lots of people in the general population that have these small changes and they do not impact on them in any way”*. In other cases, variants of unknown significance were explained to prospective adopters as inherently uncertain (table 3.1b).

#### Genetic testing can assist adoption

Although some social workers were ambivalent about genetic testing *(“I don’t know if genetic testing is going to help me find a good home for this child”*), many believed that it can assist adoption by removing uncertainty about the child’s health. They explained that most adopters were averse to uncertainty, and described situations in which an impending genetic investigation had *“put off”* adopters. For these reasons, information was considered to be beneficial to adoption. One social worker described genetic testing as simply *“more information”* (table 2.3) with which to alleviate adopters’ concerns.

These views were sharply contrasted by the medical advisors who were concerned that adopters could be misled into thinking that testing would necessarily yield clear and definitive results (table 3.1b). An argument frequently made by adoption services is that genetic testing will make it easier to place a child with a family. However, one medical advisor countered this view (table 3.3)

### ‘Genetic testing can confirm or exclude a specific aetiology’

Many social workers claimed that genetic testing could be used to confirm or exclude a specific aetiology. Clinical utility was overestimated because it was assumed that genetic causes were singular and that detection was straightforward. An illustration of this was provided by one senior social worker: “*I think it’s easier when you’re trying to confirm a specific thing actually, because you’re looking for a very specific issue which you can then rule out or in*” (table 2.2a) While this applies to the second category of genetic assessment (see above), the metaphor of *ruling in* or *ruling out* a genetic condition does not apply well to the first category; social workers were inclined to oversimplify causality as either genetic or non-genetic.

#### Genetic testing can confirm or exclude a diagnosis if there is a family history of a genetic condition

Referrals for genetic testing may arise if a child is presenting with problems alongside a family history of a (suspected) genetic condition. However, social workers reported several cases in which diagnostic testing of unaffected children had occurred on the basis of family history alone. One social worker described a case in which adopters had pursued genetic testing because a chromosomal abnormality had been detected in an older sibling. Against the recommendations of the medical advisor, *“the little girl was eventually tested and indeed she did have the same chromosome issue as her older sister”*.

All the medical advisors we interviewed reported cases involving inappropriate requests for genetic testing. One advisor described how a social worker had initiated a court order to test an unaffected child based on unverified information that the birth father had Noonan syndrome. Another advisor described a similar case involving a family history of Charcot-Marie-Tooth. Implicit to these requests was the assumption that testing for a family mutation would confirm or exclude a genetic diagnosis in the child (table 3).

#### Genetic testing can confirm or exclude a diagnosis of FASD

Social workers indicated that FASD affecting looked-after children was a growing concern. For children presenting with non-specific dysmorphic features, establishing a definitive diagnosis was considered important for assessment and adoption. One social worker believed that genetic testing could be used to diagnose FASD (table 2.2b).

Microarray testing was described by several medical advisors as a procedure for excluding chromosomal abnormalities in order to assist diagnosis. Although they insisted that genetic testing should not replace clinical judgement, one medical advisor indicated that social workers were misled in thinking that there was a test for FASD (table 3). Another medical advisor explained that, because microarray was used as a kind of ‘negative test’ for FASD, there was a tendency to overuse it in the clinic: “*I think if the case is strong history of using alcohol heavily during pregnancy, if the child is showing from birth some indication that he or she would be foetal alcohol syndrome … why should I do an array CGH?*”

## Discussion

The study found that social workers often described ‘genetic testing’ as a generic procedure for detecting the presence or absence of a specific aetiology, the results of which were believed to be definitive and mutually exclusive. In the absence of specific knowledge about genetic testing, social workers adopted an essentialist model (a sociocognitive heuristic that assumes simple causality and gene determinism) to make sense of past cases.^21^ Although some displayed knowledge of gene expression and gene-environment interaction, social workers assumed that genetic testing offered clarity in adoption because it removed uncertainty about the child’s health. Many explained that these uncertainties were unpalatable to a majority of adopters.

It was not unexpected to find that medical advisors were better informed about genetic testing and therefore recognised that many genetic conditions are multifactorial. Indeed, they were far more circumspect and agnostic about the outcomes of genetic testing, especially the results of aCGH. Rather than reducing uncertainties about the child, many advisors explained that uncertainty was an unavoidable outcome of assessment, and often at odds with the knowledge and expectations of social workers and prospective adopters. Cases that involved reporting variants of unknown significance were described as unwelcome outcomes that required careful and sensitive communication.

Medical advisors also reported multiple cases involving inappropriate requests for testing from social workers, special guardians, prospective adopters and legal professionals. There was apparently a general misconception that genetic testing was capable of confirming or excluding a genetic condition in the child. In some cases, testing was pursued for unaffected children on the basis of family history. Medical advisors expressed concerns that testing unaffected children was medically and ethically inappropriate as well as an unsuitable strategy for diagnosing complex, multifactorial conditions.

The study also revealed cases in which social workers and legal professionals believed in the existence of a genetic test for FASD. This may arise from the fact that some medical advisors use microarray to exclude chromosomal abnormalities in children presenting with dysmorphic features. However, several medical advisors stressed that diagnostic testing for FASD should be approached with caution. Where there is clinical suspicion of FASD, children should receive formal clinical assessment as well as detailed assessment of the available family before deciding to pursue a genetic investigation. Even then, the outcomes may have limited clinical utility and may affect the child’s prospects of adoption. A negative aCGH result could be misinterpreted as confirming a diagnosis of FASD, while finding a microdeletion of reduced penetrance could be taken inappropriately as undermining the same diagnosis.

## Conclusion

This study explores the ways in which different professional groups make sense of genetic assessment in adoption. Social workers and medical advisors play a crucial role in ensuring that vulnerable children are properly assessed and supported to achieve permanency with an adoptive family. However, recent advances in genetic technologies, notably the shift from genetic to genomic testing, are changing the ways in which professionals understand and tolerate uncertainty. This study reveals cases in which social workers and medical advisors have different understandings and expectations about the clinical utility of genetic testing. The priority for social workers is to use health information to increase the transparency of information about the child in order to secure their placement with a suitable family. In many cases, information regarding medical and developmental uncertainties is construed as obstacles to the adoption process. The priority for medical advisors is to report these uncertainties as and when they apply, to place the risks of the child in context and to protect the child’s future autonomy from unnecessary testing. The findings suggest that social workers would benefit from specific training on the ethical and technical aspects of genetic and genomic testing, while multidisciplinary team meetings would provide a practical forum to discuss the contrasting priorities of adoption professionals.
